# Hepatitis B vaccination of preterm infants and risk of bronchopulmonary dysplasia: a cohort study, Australia

**DOI:** 10.2471/BLT.24.291683

**Published:** 2025-01-31

**Authors:** Hannah J Morgan, Marcel F Nold, Gonzalo Sepulveda Kattan, Diana Vlasenko, Atul Malhotra, James H Boyd, Hazel J Clothier, Jim P Buttery

**Affiliations:** a Department of Paediatrics, University of Melbourne, Royal Children’s Hospital, 50 Flemington Road, Parkville 3052, Victoria, Australia.; b Department of Paediatrics, Monash University, Melbourne, Australia.; c Epidemiology Informatics, Centre for Health Analytics, Melbourne Children’s Campus, Melbourne, Australia.; d Department of Public Health, La Trobe University, Melbourne, Australia.

## Abstract

**Objective:**

To determine whether hepatitis B virus (HBV) vaccination of extremely preterm infants (defined in our study as < 29 weeks gestation) within 24 hours of birth (birth-dose) increases the risk of developing bronchopulmonary dysplasia.

**Methods:**

Using data from Australia, we conducted a population data linkage study using the Victorian Vaccine Safety Health Link. This platform links state-wide immunization and health outcomes from the Victorian Perinatal Data Collection and the Victorian Admitted Episodes Dataset. Our retrospective cohort study included all extremely preterm infants born alive during 2017–2020 (excluding data outliers). We investigated the relationship between birth-dose HBV vaccination and bronchopulmonary dysplasia diagnosis at 36 weeks postmenstrual age. We identified possible confounders using a directed acyclic graph, and included these confounders in a robust Poisson regression model.

**Findings:**

Of the 818 extremely preterm infants meeting our inclusion criteria, 306 received birth-dose HBV vaccination: 50.7% (155/306) of the vaccinated and 61.9% (317/512) of the unvaccinated infants developed bronchopulmonary dysplasia. After accounting for measured confounders, the adjusted relative risk was 0.83 (95% confidence interval, CI: 0.68–1.00), suggesting no increased risk. However, residual confounding by indication may still be present as it is not known how clinician perception of the stability of the newborn affects the decision to vaccinate or not, potentially underestimating any association between vaccination and outcome.

**Conclusion:**

Our findings support existing World Health Organization recommendations to immunize all infants against HBV within 24 hours of birth, including those born prematurely.

## Résumé

**Objectif:**

Déterminer si la vaccination contre le virus de l’hépatite B (VHB) des grands prématurés (définis dans notre étude comme nés à moins de 29 semaines de gestation) dans les 24 heures suivant la naissance (dose à la naissance) augmente le risque de développer une dysplasie bronchopulmonaire.

**Méthodes:**

Nous nous sommes basés sur des données australiennes pour mener une étude de couplage de données sur la population en utilisant le Vaccine Safety Health Link de l’État de Victoria. Cette plateforme relie les résultats en matière d’immunisation et de santé à l’échelle de l’État à partir des registres Victorian Perinatal Data Collection (collection de données périnatales) et Victorian Admitted Episodes Dataset (ensemble de données sur les épisodes admis). Notre étude de cohorte rétrospective a inclus tous les grands prématurés nés vivants au cours de la période 2017–2020 (à l’exclusion des données aberrantes). Nous avons étudié la relation entre la vaccination contre le VHB à la dose de naissance et le diagnostic de dysplasie bronchopulmonaire à 36 semaines d’âge post-menstruel. Nous avons identifié les facteurs de confusion possibles à l’aide d’un graphe acyclique dirigé et nous avons inclus ces facteurs de confusion dans un modèle robuste de régression de Poisson.

**Résultats:**

Sur les 818 grands prématurés répondant à nos critères d’inclusion, 306 ont reçu une dose de vaccin à la naissance contre le VHB : 50,7% (155/306) des nourrissons vaccinés et 61,9% (317/512) des nourrissons non vaccinés ont développé une dysplasie bronchopulmonaire. Après prise en compte des facteurs de confusion mesurés, le risque relatif ajusté était de 0,83 (intervalle de confiance (IC) à 95%: 0,68–1,00), ce qui suggère l’absence d’augmentation du risque. Cependant, il se peut que des facteurs de confusion résiduels liés à l’indication soient encore présents, car nous ne savons pas comment la perception par le clinicien de la stabilité du nouveau-né affecte la décision de vacciner ou non, situation qui pourrait sous-estimer toute association entre la vaccination et l’issue de la maladie.

**Conclusion:**

Nos résultats soutiennent les recommandations actuelles de l’Organisation mondiale de la santé de vacciner tous les nourrissons contre le VHB dans les 24 heures suivant la naissance, y compris les prématurés.

## Resumen

**Objetivo:**

Determinar si la vacunación contra el virus de la hepatitis B (VHB) de los neonatos extremadamente prematuros (definidos en este estudio como <29 semanas de gestación) en las 24 horas siguientes al nacimiento (dosis al nacimiento) aumenta el riesgo de desarrollar displasia broncopulmonar.

**Métodos:**

A partir de datos de Australia, se realizó un estudio de vinculación de datos poblacionales a través del Victorian Vaccine Safety Health Link. Esta plataforma vincula la inmunización en todo el Estado y los resultados sanitarios de la Victorian Perinatal Data Collection y el Victorian Admitted Episodes Dataset. En el estudio de cohorte retrospectivo, se incluyó a todos los neonatos extremadamente prematuros nacidos vivos durante 2017 y 2020 (excluyendo los datos atípicos). Se investigó la relación entre la dosis de vacunación contra el VHB al nacer y el diagnóstico de displasia broncopulmonar a las 36 semanas de edad posmenstrual. Se identificaron posibles factores de confusión mediante un gráfico acíclico dirigido y se incluyeron en un modelo robusto de regresión de Poisson.

**Resultados:**

De los 818 neonatos extremadamente prematuros que cumplían los criterios de inclusión, 306 recibieron dosis de vacunación contra el VHB al nacer. Se observó que el 50,7% (155/306) de los neonatos vacunados y el 61,9% (317/512) de los no vacunados desarrollaron displasia broncopulmonar. Tras tener en cuenta los factores de confusión medidos, el riesgo relativo ajustado fue de 0,83 (intervalo de confianza del 95%, IC: 0,68-1,00), lo que sugiere que no se observó un aumento del riesgo. Sin embargo, aún puede haber confusión residual por indicación, ya que se desconoce cómo afecta la percepción del médico sobre la estabilidad del neonato a la decisión de vacunar o no, lo que podría subestimar cualquier asociación entre la vacunación y el resultado.

**Conclusión:**

Los resultados obtenidos respaldan las recomendaciones existentes de la Organización Mundial de la Salud de inmunizar a todos los neonatos contra el VHB en las 24 horas siguientes al nacimiento, incluidos los prematuros.

## ملخص

الغرض تحديد ما إذا كان تطعيم فيروس التهاب الكبد الوبائي ب (HBV) للأطفال المبتسرين للغاية (المحدد في دراستنا على أنه أقل من 29 أسبوعًا من الحمل)، في غضون 24 ساعة من الولادة (جرعة الولادة)، يزيد من خطر الإصابة بخلل التنسج القصبي الرئوي.

الطريقة باستخدام البيانات من أستراليا، أجرينا دراسة ربط بيانات السكان باستخدام رابط صحة وسلامة اللقاحات الفيكتورية. تربط هذه المنصة بين التحصين على مستوى الولاية، والنتائج الصحية من تجميع بيانات ما قبل وبعد الولادة الفيكتورية، ومجموعة بيانات حالات القبول الفيكتورية. شملت دراستنا الأترابية بأثر رجعي جميع الأطفال المبتسرين للغاية، الذين ولدوا أحياء خلال الفترة 2017 إلى 2020 (باستثناء القيم المتطرفة للبيانات). لقد حققنا في العلاقة بين لقاح فيروس التهاب الكبد الوبائي ب في جرعة الولادة، وتشخيص خلل التنسج القصبي الرئوي في عمر 36 أسبوعًا بعد الولادة. حددنا عوامل التداخل المحتملة باستخدام رسم بياني غير دوري موجه، وأدرجنا هذه العوامل في نموذج التحوف لبواسون.

النتائج من بين 818 رضيعًا مبتسرًا للغاية يستوفون معايير الإدراج لدينا، تلقى 306 رضيعًا جرعة الولادة بلقاح فيروس التهاب الكبد الوبائي ب: %50.7 (155/306) من الرضع المطعمين، و%61.9 (317/512) من الرضع غير المطعمين، أصيبوا بخلل التنسج القصبي الرئوي. بعد احتساب عوامل التداخل المقاسة، كان الخطر النسبي المعدل 0.83 (بنسبة أرجحية مقدارها %95: 0.68 إلى 1.00)، مما يشير إلى عدم وجود خطر متزايد. ومع ذلك، قد لا يزال التداخل المتبقي حسب الإشارة موجودًا لأنه من غير المعروف كيف يؤثر تصور الطبيب لاستقرار المولود على قرار التطعيم من عدمه، مما قد يقلل من تقدير أي ارتباط بين التطعيم والنتيجة.

الاستنتاج تدعم نتائجنا التوصيات الحالية لمنظمة الصحة العالمية الهادفة لتحصين جميع الأطفال ضد فيروس التهاب الكبد الوبائي ب خلال 24 ساعة من الولادة، بما في ذلك الأطفال الذين يولدون قبل الأوان.

## 摘要

**目的:**

确定极早产儿（在本研究中定义为胎龄小于 29 周）在出生后 24 小时内接种乙型肝炎病毒 (HBV) 疫苗（出生后首剂疫苗）是否会增加其出现支气管肺发育不良的风险。

**方法:**

利用澳大利亚的数据，我们借助维多利亚州疫苗安全健康链接进行了一项人口数据链接研究。该平台将维多利亚州围产期数据收集和维多利亚州入院事件数据集中的全州免疫接种和健康结果进行了链接。我们对 2017 年至 2020 年间所有存活的极早产儿（不包括异常数据）进行了回顾性队列研究。我们研究了出生后接种首剂 HBV 疫苗与矫正胎龄 36 周时出现支气管肺发育不良诊断结果之间的关系。我们使用有向无环图确定了可能的混杂因素，并将这些混杂因素纳入稳健的泊松回归模型。

**结果:**

在符合纳入标准的 818 名极早产儿中，有 306 名出生后接种了首剂 HBV 疫苗：50.7% (155/306) 接种疫苗的婴儿和 61.9% (317/512) 未接种疫苗的婴儿出现了支气管肺发育不良情况。在考虑了已测量的混杂因素后，调整后的相对风险为 0.83（95% 置信区间 (CI)：0.68-1.00），表明风险没有增加。然而，由于尚不清楚临床医生对新生儿稳定性的看法会如何影响是否为其接种疫苗的决定，因此可能仍然存在适应症方面的残余混杂，这导致有可能低估了疫苗接种与结果之间的任何关联。

**结论:**

我们的研究结果支持世界卫生组织现有的建议，即在出生后 24 小时内为所有婴儿（包括早产婴儿）接种 HBV 疫苗。

## Резюме

**Цель:**

Определить, повышает ли риск развития бронхолегочной дисплазии вакцинация против вируса гепатита В (HBV) экстремально недоношенных детей (в рамках исследования с продолжительностью беременности менее 29 недель) в течение 24 часов после рождения (доза вакцины, вводимая ребенку при рождении).

**Методы:**

На основании данных, полученных в Австралии, было проведено исследование взаимосвязи между демографическими показателями с использованием системы Victorian Vaccine Safety Health Link. Данная платформа связывает данные об иммунизации и состоянии здоровья иммунизированных лиц в масштабах государства, полученные в результате сбора перинатальных данных штата Виктория и данных о случаях госпитализации в штате Виктория. В проведенное ретроспективное когортное исследование вошли все экстремально недоношенные младенцы, родившиеся живыми в период 2017–2020 годов (за исключением выпадающих значений). Была изучена связь между введением ребенку при рождении дозы вакцины против гепатита В и постановкой диагноза бронхолегочной дисплазии на 36-й неделе после дня последней менструации у матери. С помощью направленного ациклического графа были определены возможные факторы, затрудняющие оценку причинно-следственной связи, которые были включены в робастную модель регрессии Пуассона.

**Результаты:**

Из 818 экстремально недоношенных младенцев, отвечающих критериям включения, 306 получили при рождении дозу вакцины против гепатита В: у 50,7% (155/306) вакцинированных и 61,9% (317/512) невакцинированных младенцев развилась бронхолегочная дисплазия. После учета измеренных факторов, затрудняющих оценку причинно-следственной связи, скорректированный относительный риск составил 0,83 (95%-й доверительный интервал, ДИ: 0,68–1,00), что свидетельствует об отсутствии повышенного риска. Тем не менее все еще может присутствовать остаточное искажение по показаниям, поскольку неизвестно, как восприятие врачом стабильности состояния новорожденного влияет на решение о вакцинации или отказе от нее, что может привести к искажению оценки любой связи между вакцинацией и результатом.

**Вывод:**

Полученные результаты подтверждают существующие рекомендации Всемирной организации здравоохранения по иммунизации всех младенцев против вируса гепатита В в течение 24 часов после рождения, включая недоношенных.

## Introduction

Hepatitis B virus (HBV) is the most common blood-borne virus in Australia, with an incidence of up to 61 per 100 000 per year in the age group 30–39 years.^1^ HBV can be transmitted by inoculation through broken or penetrated skin, or by mucosal contact with blood and other bodily fluids from an infected person, including vertically from mother to infant during pregnancy and birth.^2^ HBV infects the liver to cause an acute infection phase and/or a chronic infection phase, which can potentially lead to cirrhosis and hepatocellular carcinoma with significant liver-related mortality (820 000 premature deaths, i.e. before life expectancy, globally in 2019).^2^^,^^3^ Infants infected at or soon after birth are 90% more likely to develop chronic infection compared with those infected at older ages.^2^^,^^4^

The most effective preventive intervention for HBV infection is vaccination. The successful implementation of universal infant HBV vaccination, with the first dose shortly after birth, has been key to reducing HBV infection and disease globally. For high-risk newborns of infected mothers, this primary vaccine alongside administration of hepatitis B immunoglobulin at birth protects 94–99% of infants.^5^^,^^6^ The Australian public health agency and the Pan American Health Organization provide guidelines recommending a four-dose HBV vaccination schedule that includes preterm infants (< 37 weeks gestation),^7^ with doses given within 24 hours of birth, and at the age of 6 or 8 weeks, 4 months and 6 months.^8^^,^^9^ In some hospitals in China, Macau Special Administrative Region, guidelines recommend all infants, including preterm infants, to receive the first dose of the HBV vaccine at birth.^10^ An additional fifth booster dose at the age of 12 months is recommended for those born before 32 weeks gestation and/or infants with a low birth weight (< 2000 g), as these infants usually mount a lower serologic response to HBV vaccination.^11^^–^^13^ Two studies show that in settings where a birth dose is recommended, attaining high birth-dose coverage in preterm infants is feasible.^14^^,^^15^ However, it is unknown from these studies whether the birth doses were correctly administered within the first 24 hours, because of the possibility that more unwell babies may have their vaccination delayed.^14^^,^^15^

The safety of the birth-dose HBV vaccination in newborn infants weighing 2000 g or more is well established.^6^^,^^16^ However, due to limited evidence on its safety in preterm infants with birth weight less than 2000 g, most national immunization schedules recommend withholding the birth-dose vaccination for this group.^17^

The chronic lung disease bronchopulmonary dysplasia is one of the most important complications of prematurity, contributing to substantial short- and long-term morbidity.^18^ A recent prospective immune profiling study conducted on Australian extremely preterm infants (defined in this study as < 29 weeks gestation) observed a strong association between T-helper lymphocyte type 2 (Th2) immune polarization (using the surrogate marker interleukin-4, IL-4+, in T-cells) from birth and bronchopulmonary dysplasia. There was also a positive association between early HBV vaccination and a higher IL-4+ T-cell percentage.^19^ These data suggested the possibility of an increased risk of the disease as a result of early HBV vaccination driving Th2 immune polarization and type 2 cytokine activation in infants born before 32 weeks gestation.

Here we aim to determine whether birth-dose HBV vaccination in extremely preterm infants increases their risk of bronchopulmonary dysplasia. 

## Methods

The Australian Department of Health reported that “chronic lung disease is the single most important factor determining length of hospital stay in babies born at less than 29 weeks”;^20^ we therefore use this definition (and not that of the World Health Organization (WHO), which is < 28 weeks) for extremely preterm infants in our study. Because randomized controlled trials do not provide an ethical or feasible way to answer this question, we explored the optimal utilization of real-world population-level data sourced from two different state-based data sets of the Department of Public Health, Victoria. Our cohort study included all extremely preterm infants born alive in Victoria from 1 January 2017 to 31 December 2020.

### Data sources

Surveillance of Adverse Events Following Immunization in the Community is the central vaccine safety surveillance service for Victoria, Australia. To ensure the continued safety of vaccines after approval, the surveillance service collects data on vaccine adverse events in the community via a combination of active and spontaneous methods and, more recently, using linked data. The linked data platform, the Vaccine Safety Health Link, was used for this study. The platform is the service’s repository of anonymously linked immunization, hospital, mortality, perinatal and notifiable disease records for every resident in Victoria.^21^ These records contain demographic and medical information to enable a rapid and sensitive investigation of vaccine safety concerns, and with a lower data bias compared to surveillance methods that rely exclusively on reporting.^21^

We acquired data from the Victorian Perinatal Data Collection and the Victorian Admitted Episodes Dataset, of which the Department of Health, Victoria is the custodian.^22^^,^^23^ The former includes data on maternal and perinatal outcomes for the Consultative Council on Obstetric and Paediatric Mortality and Morbidity, and the latter includes data on patient admissions at public and private hospitals, including rehabilitation centres, extended care facilities and day procedure centres.^22^ As part of the Vaccine Safety Health Link project, the Centre for Victorian Data Linkage linked these two data sets using a combination of deterministic and probabilistic methods. The data sets are provided in a database with de-identified unique person-identifiers attached to each record. The unique person-identifiers are linking records within and between the data sets to examine population trends before and after vaccination.

### Data acquisition

We first filtered the Victorian Perinatal Data Collection for relevant infants and extracted birth-dose HBV vaccination status and other variables (number of weeks gestation; birth weight in grams; Apgar score of health at 1 and 5 minutes; Aboriginal and/or Torres Strait Islander indigenous status; maternal age; mothers’ smoking status; and congenital heart disease status). For the purposes of this study, we considered the absence of HBV vaccination in the first 24 hours after birth as unvaccinated, irrespective of any other vaccinations given during this time period, or HBV vaccination given after the 24-hour period. 

We determined the bronchopulmonary dysplasia status (outcome) of each extremely preterm infant from the Victorian Admitted Episodes Dataset according to the presence or absence of a P27.1, P27.8 or P27.9 *International Statistical Classification of Diseases and Related Health Problems, Tenth Revision* (ICD-10) Australian modification code in one of the 40 available diagnosis fields. Because it is not possible to diagnose the disease in infants with a postmenstrual age of less than 36 weeks, these ICD-10 Australian modification codes are assigned if the infant still requires respiratory support at this age. 

We merged vaccination and outcome status from the different data sets based on the unique person-identifier to create a single data set for our study. We excluded records with data entry errors or outliers with more than three standard deviations from the mean in birth weight. We considered records where the number of weeks of gestation was reported as 20 weeks or less as data entry errors, which we also excluded from our analysis.

### Data processing

We analysed the combined data set using the sandwich package of R, version 4.3.1 (R Foundation, Vienna, Austria).^24^^,^^25^ We identified possible confounders in consultation with neonatologists using a directed acyclic graph, which provides a visual representation of potentially meaningful relationships between variables related to the research question (available in online repository).^26^^,^^27^ We compared summary statistics of baseline health characteristics between extremely preterm infants who were vaccinated and unvaccinated to birth-dose HBV vaccination. Finally, we analysed the outcome of bronchopulmonary dysplasia using a robust Poisson regression model, including HBV vaccination exposure and confounders as covariates.

We conducted a secondary analysis, examining all-cause mortality occurring in the 3 months after birth as the outcome. We obtained mortality status from the Victorian Deaths Index data set.

To identify any potential bias introduced by the observational nature of our study, as opposed to an approach using a randomized controlled trial, we used a target trial emulation framework (online repository).^27^^,^^28^

### Ethical considerations

The Royal Children’s Hospital Melbourne Ethics Committee oversees all work conducted as part of the Vaccine Safety Health Link project (no. 79964).

## Results

During the study period, 10 029 infants were born preterm, 9188 of which were born after 29–36 weeks gestation and 17 of which were stillborn, leaving 824 extremely preterm infants who were born alive. We considered that the records for six of these infants included data outliers, and we conducted our analysis on the remaining 818 infants.

Of the 818 infants in the study, 306 received the birth-dose HBV vaccination and 512 did not. We noted that all indicators of baseline health were consistent between these birth-dose vaccinated and unvaccinated groups (Table 1). At the age of 24 months, 785 (96.0%) had received at least one dose of the HBV vaccination.

**Table 1:** Comparison of key factors between birth-dose HBV vaccinated and unvaccinated preterm infants, Australia, 2017–2020

Factor	Vaccinated (*n* = 306)	Unvaccinated (*n* = 512)	*P* ^a^
No. with indigenous status (%)	6 (2.0)	22 (4.1)	0.10
Median maternal age, years (IQR)	32 (7)	33 (8)	0.05
Median gestation, weeks (IQR)^b^	27 (2)	27 (3)	1.00
Median Apgar score at 1 minute (IQR)	5 (3)	5 (4)	1.00
Median Apgar score at 5 minutes (IQR)	8 (3)	8 (3)	1.00
No. with congenital heart disease (%)	3 (0.9)	7 (1.3)	0.87
No. deaths in first 3 months (%)	7 (2.3)	14 (2.7)	0.87
Median weight, g (IQR)^b^	918 (371)	889 (330)	0.92

IQR: interquartile range.

^a^ *P*-value ≤ 0.05 indicates a statistically significant difference between the vaccinated and unvaccinated infants.

^b^ Boxplots are provided in online repository.^27^

We observed that 50.7% (155/306) of the vaccinated infants and 61.9% (317/512) of the unvaccinated infants developed bronchopulmonary dysplasia. This outcome yields an unadjusted relative risk (RR) of 0.81 (95% confidence interval, CI: 0.67–0.98), with no evidence of an increased risk of the development of the disease following birth-dose HBV vaccination.

We identified measurable potential confounders from the directed acyclic graph and pertinent data, including maternal age, low Apgar at 1 minute, low Apgar at 5 minutes, maternal smoking, gestation period and congenital heart disease status (online repository).^27^ After adding these confounders to the robust Poisson model, we obtained a RR of 0.83 (95% CI: 0.68–1.00), again indicating no increased risk of the development of the disease following birth-dose HBV vaccination. 

Our secondary analysis investigating all-cause mortality in the first 3 months of life as the outcome found that 2.3% (7/306) of the vaccinated group and 2.7% (14/512) of the unvaccinated group died during this period. These results show no increased risk of mortality following birth-dose HBV vaccination (unadjusted RR: 0.83; 95% CI: 0.32–2.00). After maternal age, we included low Apgar at 1 minute, low Apgar at 5 minutes, maternal smoking and congenital heart disease status as potential confounders to the model; we observed a RR of 1.13 (95% CI: 0.42–2.81), showing no evidence of an increased risk of mortality following birth-dose HBV vaccination.

## Discussion

In this Australian cohort, we identified no evidence of an increased risk of bronchopulmonary dysplasia in extremely preterm infants who received birth-dose HBV vaccination in the first 24 hours of life, supporting the continued use of birth-dose HBV vaccination in the extremely preterm population.^16^^,^^29^ As paediatric health researchers affiliated to Australian institutes, we are uniquely placed to conduct this research: Australia has one of the few health-care systems that routinely recommend birth-dose HBV vaccination in all infants, including those born preterm and with birth weight less than 2000 g.^10^ However, despite these national recommendations, the proportions of birth-dose HBV vaccination of extremely preterm infants appear to vary between Victorian neonatal units. Currently, the state of Victoria is the only Australian jurisdiction with statewide data-linking vaccination records, birth records, and perinatal and infant outcomes. Ultimately, our data set was able to capture over 800 extremely preterm newborns born over a 4-year period.

The initial concerns were raised by Australian researchers who observed an association between Th2 immune polarization and an increased risk of bronchopulmonary dysplasia.^19^ The same prospective study sampling between birth and age 16 weeks in both extremely preterm and full-term infants found an association between birth-dose HBV vaccination and Th2 polarization. In animal models of neonatal cardiopulmonary illness, blockage of Th2 cytokines led to amelioration of lung inflammation and protection of alveolar and vascular integrity.^30^ These concerns were further supported by data showing that alum salts used as vaccine adjuvants, including in HBV vaccination, may potentiate serologic responses, inducing a Th2-biased cellular immune response.^31^ Such responses may contribute to the pathogenesis of bronchopulmonary dysplasia and other chronic lung diseases.^32^

The incidence of bronchopulmonary dysplasia increases with decreasing gestational age at birth; diagnosis in premature infants brings an increased risk of in-hospital and out-of-hospital mortality in the first year of life, with mortality up to around 50% when accompanied by associated pulmonary hypertension.^33^^–^^35^ The disease also affects multiple organ systems, with long-term morbidity described in cohort studies of children aged up to 11 years including increased risk of asthma, impaired cardiac function and neurodevelopmental disability.^34^ As the current Australian national recommendations are to immunize all infants against HBV at birth, regardless of gestation or weight, addressing whether there was a positive association between a birth dose and a subsequent diagnosis of bronchopulmonary dysplasia was a national priority.

The primary strengths of our study are the population-based cohort design and large sample size of real-world data that enabled quantification of the risk of bronchopulmonary dysplasia in extremely preterm infants following birth-dose HBV vaccination. We were able to limit the risk of bias during the emulation stage and adjust for some important confounders (e.g. maternal age, maternal smoking, Apgar score and congenital heart disease status)^36^ by using a linkage approach for two large and varied independent national data sets.

Our study had several limitations, including an inability to control for confounding variables in several other areas. First, the use of secondary data did not allow for data collection of other potentially confounding variables identified in the directed acyclic graph, including infections and the type of respiratory support administered (online repository).^27^ Second, clinician perception of the overall clinical stability of the newborn may affect the decision to vaccinate at birth, with newborn infants perceived as more unwell on their first day of life (particularly those with bacteraemia and respiratory support) being less likely to receive birth-dose vaccine. If true, decisions to withhold vaccination could lead to an underestimation of the association and mask the increased risk of bronchopulmonary dysplasia as a result of birth-dose HBV vaccination through confounding by indication. Third, data on the sex of the infants were unavailable; some evidence suggests that extremely preterm male infants may experience greater respiratory morbidity, so a lack of sex-disaggregated data analysis may be masking any increased male risk of birth-dose HBV vaccination.^37^^,^^38^ Fourth, different administration behaviour by clinicians may vary between neonatal intensive care units across Victoria, and we were not able to discern in which of the five tertiary neonatal units in greater Melbourne each infant received treatment. HBV vaccine administration practices may vary between these units according to their scope of practice (e.g. only two of the five units offer surgical treatment) and, to some degree, patient clientele in terms of ethnicity, socioeconomic status or pregnancy complications. Unmeasured confounding in these four areas is likely to be the main reason for the differences in results between our study and another published study.^19^

Another limitation is that vaccination data for infants within the first 24 hours of life and before discharge are best captured in the Victorian Perinatal Data Collection, and vaccination data for infants after the first week of life are best captured by the Australian Immunisation Register, leaving a potential gap in accurate records. However, compliance with non-birth-dose HBV vaccination is high, with almost all included infants receiving at least one dose by the age of 24 months. Because of the gap in records, we were unable to assess the relationship between vaccination administered in the first week of life and the incidence of bronchopulmonary dysplasia. 

The lack of HBV vaccine brand information was a limitation since we could not perform an analysis by brand. Despite this, there is no reason to believe the results would vary by brand, considering both hepatitis B monovalent vaccines Engerix-B and H-B-Vax II available in Australia contain alum salt adjuvants, namely aluminium hydroxide hydrate and aluminium hydroxyphosphate sulfate, respectively.^39^^,^^40^

A further limitation of our study is that secondary observational data have the potential to introduce measurement bias associated with systematic issues with inaccurate data recording. The use of de-identified secondary data meant that we were unable to access source records to confirm whether episodes with ICD-10 (Australian modification) codes for bronchopulmonary dysplasia adequately fulfilled case definition criteria for these conditions or determine severity; defining and diagnosing the disease is notoriously challenging and not always reliably undertaken, despite best intentions.^41^ Extremely preterm infants who died from illness preceding what would have been bronchopulmonary dysplasia if they had survived until 36 weeks will have been listed as not having the outcome. To investigate this, we conducted a secondary analysis on the impact of HBV vaccination on all-cause mortality in extremely preterm infants, and found no evidence of an increased risk of mortality. Noteworthy is that the cause of death for most of these infants appeared to be unrelated to the disease and multifactorial in nature, with most deaths probably a result of prematurity or congenital abnormalities (i.e. preceding the HBV vaccination) and not relevant to our assessment. Future analyses with source-note review and collection of information around the vaccination, outcome and confounders, including the diagnosis of the disease and the circumstances leading to mortality, would account for some of the limitations introduced from the use of routinely collected data.

There exists high heterogeneity in HBV vaccination schedules among extremely preterm infants between different countries and regions. In a systematic review, we found that the guidelines in only two out of 15 countries and areas included the strategy recommended by WHO of giving the nationwide first dose as soon as possible after birth (ideally within 24 hours), regardless of gestational age or body weight.^10^ Another 40% (6 countries and areas) recommend waiting until the infant reaches a determined milestone (weight of 2000–2200 g, or chronological age of 1 month or hospital discharge, whichever comes first) and the remaining 47% (7 countries and areas) have varying guidelines or no unified recommendation. 

The recommended guidelines and the clinical perception of risk associated with birth-dose HBV vaccination may cause variation in vaccine administration among extremely preterm and full-term infants. Addressing the question about the timing of initial HBV vaccination within 24 hours of birth for the extremely preterm infant population, our study suggests that there is no evidence of any additional harm or risk of bronchopulmonary dysplasia using the birth dose for these neonates. Although only a large multicentre randomized controlled trial can definitively account for all potential confounding, our findings provide support to existing WHO recommendations to immunize all infants against HBV within 24 hours of birth.
